# Personality Profiles and Psychological Adjustment in Breast Cancer Patients

**DOI:** 10.3390/ijerph17249452

**Published:** 2020-12-17

**Authors:** M. Victoria Cerezo, María J. Blanca, Marta Ferragut

**Affiliations:** Department of Psychobiology and Methodology of Behavioral Science, Faculty of Psychology, University of Malaga, Campus Universitario de Teatinos, s/n, 29071 Malaga, Spain; blamen@uma.es (M.J.B.); mferragut@uma.es (M.F.)

**Keywords:** breast cancer, clinical syndromes, MCMI, personality, psychological profile, well-being

## Abstract

Dispositional personality characteristics may play a role in psychosocial adjustment to any disease, including cancer. Purpose: The purpose of this study is to identify personality profiles in breast cancer patients and to determine whether these profiles are associated with psychological adjustment or psychopathology. Methods: Participants were 109 women (mean age, 52.01) diagnosed with breast cancer. They completed the Millon Clinical Multiaxial Inventory-III (MCMI-III), the Life Orientation Test-Revised (LOT-R), the Satisfaction with Life Scale, and the Positive and Negative Affect Scales. Results: The analysis revealed two different personality profiles: (a) one group, comprising 38.23% of the sample, was characterized by paranoid, negativistic, and dependent personality traits and was considered as a “vulnerable group”; and (b) another group (61.77%) was characterized by compulsive, histrionic, and narcissistic personality traits and was considered as a “psychologically adjusted group”. The vulnerable group scored higher than the psychologically adjusted group on all clinical syndromes, with scores above 60 on the anxiety, somatoform, dysthymic, and bipolar scales (score on anxiety being above 75); in contrast, the psychologically adjusted group did not reach a base rate score of 60 on any of the clinical syndromes, showing no manifestations of psychopathology. Additionally, the vulnerable group scored lower than the psychologically adjusted group on optimism, life satisfaction, and positive affect, but higher on negative affect. Conclusions: The results suggest that personality traits could affect the psychological adjustment of breast cancer survivors. We discuss the implications of belonging to each group and highlight the importance of early identification of vulnerable women in order to facilitate clinical and psychological support.

## 1. Background

Cancer is the second most frequent disease (20.7%) in all races and ages in females, behind heart disease (21.8%) and following chronic lower respiratory diseases (6.2%). Furthermore, cancer is the top ranking disease in women between 45 and 84 years old [1]. Specifically, breast cancer was the most common malignancy in women (followed by lung cancer) in all regions of the world in 2018 [2], except for in eastern Africa where cervical cancer dominated [3]. It represents 25.2% of the total cancer incidence, with a considerable incidence rate (43.3 per 100,000) that is higher than that of any other cancer. Belgium (113.2), Luxembourg (109.3), and the Netherlands (105.9) have the top age-standardized rate in the world, above 100 per 100,000 [4]. Furthermore, it accounts for 14.7% of all cancer deaths [5]; for example, in the United States, breast cancer is the most common cancer in females and the second most common cause of cancer death in women [6]. There are multiple risk factors of breast cancer; some of them cannot be changed (e.g., being a woman, age, breast cancer family history, and genetics) but others may be changed by making healthy choices (e.g., overweight, lack of exercise, smoking, possibility of belated pregnancy, or short breastfeeding time histories) [2,6]. The role of personality in cancer risk has been controversial, and the evidence remains inconclusive. Although there is not enough evidence to demonstrate the existence of an association with the increased risk of this illness [7], personality traits have been related to cancer in previous studies [8,9,10,11].

Cancer patients may, however, have dispositional personality characteristics that play a role in their psychosocial adjustment to the disease. In this context, a cancer diagnosis has been linked to certain personality traits related to difficulty in expressing emotions, an attitude or tendency toward helplessness or hopelessness, compliance, and focusing on other people’s needs, as well as with higher levels of anxiety and anger [8,11,12]. Research suggests, for example, that lower neuroticism is associated with better physical health and with health behaviors, whereas higher neuroticism is associated with difficulty in regulating negative emotions [9,13,14]. These personality traits are correlated with manifestations of psychopathology, and they may predispose certain cancer patients to develop disorders such as anxiety or depression during and after treatment [8,15,16,17]. Additionally, a systematic review of the literature indicates that personality traits predicted 12-month emotional distress, depression, anxiety, and trauma symptoms in breast cancer patients [17].

Conversely, there are personality traits that appear to be associated with high levels of psychosocial adjustment. The empirical evidence indicates that dispositional optimism is a strong predictor of psychosocial adjustment and subsequent quality of life among survivors, along with coping or resilience [15,16,18,19,20,21]. Breast cancer patients who scored higher on optimism reported better social, emotional, and mental functioning and better quality of life than did those who scored low on optimism [15,17,19,22,23]. Additionally, Saboonchi et al. [24] reported that initial levels of optimism inversely predicted emotional distress two years after surgery for breast cancer. These findings suggest that aspects such as optimism are related to the well-being of cancer patients. The general concept of well-being is usually considered to have both a cognitive component (life satisfaction) and an affective component (affectivity) [25].

The assessment of personality traits may contribute to a better understanding of the needs of individual patients and help to tailor psychological intervention accordingly. In the context of breast cancer units, consideration of personality profiles may be especially useful for optimizing healthcare resources. Although there are several instruments for assessing personality in clinical and nonclinical populations, only the Minnesota Multiphasic Personality Inventory (MMPI-2) and the Millon Clinical Multiaxial Inventory-III (MCMI-III) have been developed in accordance with the Diagnostic and Statistical Manual of Mental Disorders (DSM) [26]. The MCMI-III has demonstrated its usefulness for differentiating between functional and dysfunctional personality patterns across a continuum from normal prototype or personality style, traits, and clinical characteristics to disorders of personality. Its application can help to design appropriate psychological interventions [27] and it has been used with patients diagnosed with multiple sclerosis [28], cancer [29], cluster headache [30], or fibromyalgia [31]. However, studies with breast cancer patients that seek to identify common personality profiles are very scarce [29].

The purpose of this study is to extend knowledge about the identification of personality profiles among breast cancer patients and to determine whether these profiles are associated with psychological adjustment or manifestations of psychopathology. To this end, we administered the MCMI-III to 109 participants and performed a cluster analysis of the resulting data in order to identify personality profiles. We then studied the stability of the cluster solution and analyzed differences between personality profiles in relation to clinical syndromes, optimism, and well-being (life satisfaction and affectivity).

## 2. Methods

### 2.1. Participants

Participants were 109 women aged between 31 and 80 years (M = 52.01, SD = 10.80). They were all diagnosed with breast cancer, with a mean time since diagnosis of 3.28 years (SD = 3.85). The majority of participants were at stage II or IIIA of breast cancer, according to the TNM tumor classification system [32]. Table 1 shows the sample characteristics with regard to age, marital status, time since diagnosis, and cancer stage. Inclusion criteria for participants were: having a diagnosis of primary breast cancer with no other cancer diagnosis, having signed informed consent, and having provided valid data on the MCMI-III. All participants were recruited through the breast cancer association ASAMMA (Association for the Care of Women with Breast Cancer, Malaga, Spain).

### 2.2. Instruments

Personality traits and clinical syndromes. These were assessed using the Spanish version of the Millon Clinical Multiaxial Inventory-III (MCMI-III) [33]. This is a self-report questionnaire with 175 true-false items distributed across 14 personality scales (Axis-II related) and 10 clinical syndrome scales (Axis-I related). Although the MCMI-III was developed in relation to DSM-IV, the scales remain compatible with DSM-5 [34,35]. The personality scales are labeled Schizoid, Avoidant, Depressive (Melancholic), Dependent, Histrionic, Narcissistic, Antisocial, Sadistic, Compulsive, Negativistic, Masochistic, Schizotypal, Borderline, and Paranoid. The clinical syndrome scales are Anxiety, Somatoform, Bipolar (Manic), Dysthymia, Alcohol Dependence, Drug Dependence, Post-Traumatic Stress Disorder, Thought Disorder, Major Depression, and Delusional Disorder. The personality and clinical syndrome scales were scored using base rate (BR) scores, interpreted as follows: BR score between 60 and 74, presence of a trait; 75–84, clinical characteristics; 85 or above, a persistent, significant clinical concern or personality disorder. In the Spanish population, the Cronbach’s α of the MCMI-III ranges from 0.65 to 0.88. The MCMI-III has previously been used in research concerning cancer [36].

Optimism. This was assessed using the Life Orientation Test-Revised (LOT-R) [37], in its Spanish version [38]. This scale consists of 13 binary items that assess optimism as a personality disposition. High scores reveal high optimism. The scale showed adequate reliability in the present sample (α = 0.85). The LOT-R has previously been used in research concerning breast cancer [20].

Well-being was assessed in terms of:

(i) Life Satisfaction. This cognitive component of well-being was assessed using the Satisfaction with Life Scale [25], in its Spanish version [39]. The scale comprises five items, each rated on a 7-point Likert scale (1: totally disagree; 7: totally agree). High scores indicate high life satisfaction. The scale showed adequate reliability in the present sample (α = 0.77). This questionnaire has previously been used in research concerning breast cancer [40].
(ii) Affectivity. The affective component of well-being was assessed with the Positive and Negative Affect Scales, [41] in their Spanish version [42]. This instrument consists of 12 items distributed across two subscales: positive and negative affect (six items each). Each item is rated on a 5-point Likert scale (1: never; 5: always), and higher scores indicate higher levels of positive or negative affect. Both subscales showed good reliability in the present sample (α = 0.89 and α = 0.85, respectively). This questionnaire has previously been used in research concerning cancer [43].

### 2.3. Procedure

All the women participated voluntarily, providing a report of their medical history and written informed consent prior to inclusion in the study. The study was approved by the ethics committee of the University of Malaga (Spain). All procedures were performed in accordance with the ethical standards described in the 1964 Declaration of Helsinki.

All the women participated voluntarily, providing a report of their medical history and a written informed consent prior to inclusion in the study. No women refused to participate in the study. The instruments were administered on the first day that participants attended the ASAMMA. Each of the women was interviewed by an association’s psychologist, and at the end of it they completed the questionnaires in a quiet room. The psychologist informed them about the study objectives and procedures and clarified any doubts they may have had. The single session lasted around 1 h.

### 2.4. Data Analysis

The Mahalanobis distance with χ^2^ at *p* < 0.001 was used to detect multivariate outliers. In order to identify personality profiles, a cluster analysis was carried out using BR scores on the personality scales of the MCMI-III. Specifically, hierarchical agglomerative cluster analysis (average linkage) was performed, using the squared Euclidean distance as the similarity measure. The number of clusters was determined by examining the agglomeration schedule and the dendrogram. The clustering process was stopped when a large difference between the coefficients of two consecutive stages was found.

In order to determine the stability of the cluster solution, the sample was randomly split into two sub-samples, repeating the analysis with each. Subsequently, *t*-test (or Welch’s *t*-test under inequality of variances) was applied to BR scores on the personality scales in order to determine the relative contributions of the variables to the cluster solution, and hence identify their characteristics.

The clusters were also validated by comparing the groups on variables that were not included in the clustering process [44]; this was done through a series of analyses of covariance (ANCOVA). We analyzed differences between clusters in BR scores on the clinical syndrome scales of the MCMI-III and in scores on optimism, life satisfaction, and affectivity. In order to control for the effect of age, time since diagnosis, and breast cancer stage (from 0 to 6 points), these variables were considered as potential covariates. ANCOVA assesses the differences in the dependent variables after adjustment of preexisting differences in covariates so that differences between the two groups could not be attributed to differences in covariates. All analyses were performed with IBM SPSS 23 (IBM Corp. Armonk, NY, USA).

## 3. Results

### 3.1. Cluster Analysis: Personality Scales

**Figure 1:**
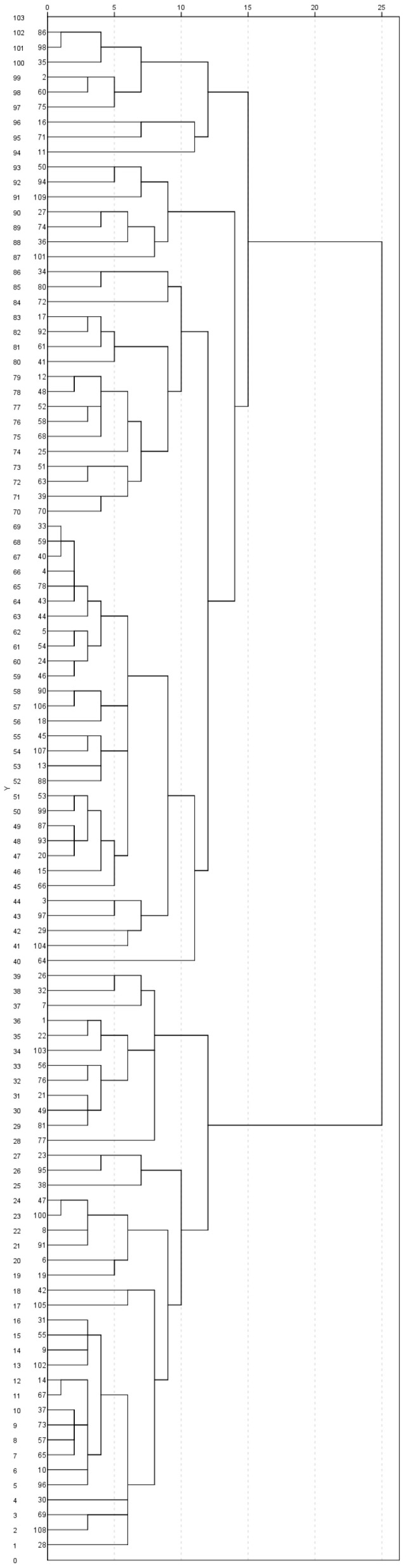
Cluster dendrogram.

Preliminary analysis identified no case as being a multivariate outlier, and hence the cluster analysis was performed with the whole sample using BR scores on the personality scales of the MCMI-III. Inspection of the agglomeration schedule and the dendrogram suggested a four-group solution with sample sizes of 39, 63, 2, and 5. The very small size of the latter two groups suggests that they may represent individuals who are very different from the rest of the sample, and hence we proceeded to repeat the analysis, excluding these two groups. The agglomeration schedule and the dendrogram of the cluster analysis with the remaining 102 participants suggested a two-group solution with sample sizes of 39 and 63, corresponding to the first two groups of the initial four-group solution (Figure 1).

In order to determine the stability of the two-group solution, the sample was randomly split into two sub-samples, repeating the analysis with each. The cluster structure in each sub-sample was similar to the full-sample solution and yielded the same two groups.

Table 2 shows the results from application of *t*-test (or Welch’s *t*-test under inequality of variances) to BR scores on the personality scales, which allow us to identify the characteristic of the two groups. All comparisons were statistically significant with high effect sizes. From a clinical point of view, it should be noted that the first group (*n* = 39) had a mean BR score above 60 on the Paranoid, Negativistic, and Dependent scales, while the second group (*n* = 63) had a mean BR score above 60 on the Histrionic, Narcissistic, and Compulsive scales.

### 3.2. Differences in Clinical Syndrome Scale Scores

The correlation between age and cancer stage with dependent variables was not statistically significant. However, the correlation between them and time since diagnosis was significant. Therefore, we performed an ANCOVA for each dependent variable with time since diagnosis as a covariate. Preliminary analysis showed that the homogeneity of regression line slopes was fulfilled for all variables. The examination of the values of skewness and kurtosis of residuals indicated that five variables showed moderate departures from the normal distribution. However, research has shown that ANCOVA as well as ANOVA is robust to violation of normality [45,46]. Thus, we proceeded with the analysis.

The results are shown in Table 3. Group 1 scored higher than group 2 on all clinical syndromes. From a clinical point of view, it should be noted that group 1 had a BR score above 60 on the Anxiety, Somatoform, Bipolar (Manic), and Dysthymia scales, with the score on Anxiety being above 75. By contrast, group 2 did not reach a BR score of 60 on any of the clinical syndromes. Therefore, group 1 was considered as a “vulnerable group” and group 2 as a “psychologically adjusted group”.

### 3.3. Differences in Optimism and Well-Being

Ten participants showed missing value in at least one of the variables considered. The Little’s test of missing completely at random (MCAR), including the two clusters and clinical syndrome scale scores, was not statistically significant; χ^2^ (58) = 70.12; *p* = 0.132, indicating that missing values were completely at random, that is to say, they randomly distributed across all observations [47]. Therefore, we proceeded with the analysis using completed cases. As in the previous case, time since diagnosis was the only variable related to the dependent variables, so we considered it as a covariate in the ANCOVA. Preliminary analysis showed that the homogeneity of regression line slopes was fulfilled and the values of skewness and kurtosis of residuals indicated that only one variable showed moderate departures from the normal distribution. The results are shown in Table 4, with all comparisons being statistically significant. The vulnerable group scored lower than the psychologically adjusted group on optimism, life satisfaction, and positive affect, but higher on negative affect. 

## 4. Discussion

The purpose of this study was to extend knowledge about the identification of personality profiles among breast cancer patients and to determine whether these profiles were associated with psychological adjustment or manifestations of psychopathology. To this end, participants completed the MCMI-III and we performed a cluster analysis of scores on the personality scales. Then, in order to study the stability of the cluster solution and to determine whether the groups differed in psychological adjustment or manifestations of psychopathology, we analyzed differences in their scores on the clinical syndrome scales of the MCMI-III and on measures of optimism, life satisfaction, and affectivity.

The results of the cluster analysis suggested two personality profiles of these breast cancer patients. The first group, formed by 38.24% of participants, showed a tendency to present paranoid, negativistic, and dependent personality traits. Thus, this group is characterized by the tendency to be suspicious, emotionally irritable, and skeptical, to regard the future with pessimism or anger, and to be compliant and conciliatory but also fragile and lacking in self-confidence [47,48]. The results from the cluster validation showed that this group is associated with a tendency toward somatoform, dysthymic, and bipolar clinical syndromes, as well as towards clinical symptoms for anxiety. It also showed a lower level of optimism, life satisfaction, and positive affect, and a higher level of negative affect. Overall, this group can be considered as a “vulnerable group” because it shows psychological maladjustment, related mainly to anxiety, and a lower level of optimism and well-being. These results are consistent with previous findings suggesting that cancer patients usually present comorbid anxiety/mood spectrum disorders [49], and also reflect the reported prevalence rate of anxiety disorder in breast cancer patients, which is around 30% [50]. This may have important clinical implications and highlights the need to enhance psychosocial support networks for these patients [12,17,29].

The second group, formed by 61.76% of participants, showed compulsive, histrionic, and narcissistic personality traits. This group is thus characterized by the tendency to be conscientious, efficient, sociable, vivacious, impetuous, animated, enthused, self-confident, optimistic, and imperturbable [47,48]. In contrast to the vulnerable group, this group did not achieve a BR score of 60 on any of the clinical syndromes and showed a higher level of optimism, life satisfaction, and positive affect and a lower level of negative affect. Based on these results, this group can be considered as a “psychologically adjusted group” because it shows an absence of psychopathology, with no clinical symptoms of anxiety, and a higher level of optimism and well-being. Some researchers have suggested that scores on the narcissistic, histrionic, and compulsive scales of the MCMI-III can be interpreted as non-pathological when clinical syndromes are not present [50,51,52]. This might correspond to what White and Gondolf [53] described as defensive “looking good” responses, a phenomenon that has been observed with different populations [30,52]. Our results also provide empirical support for the idea that optimism is an important characteristic linked to well-being in women with breast cancer [17,18,20,23]. More specifically, they are consistent with previous studies reporting that a higher level of optimism is associated with better social, emotional, and mental functioning and a better quality of life [15,17,18,19,20,21,22].

Overall, the results provide evidence that personality is a relevant factor in the psychological adjustment of cancer patients [8,9,10,11,17,50] and that it may affect the course of well-being among breast cancer survivors [29]. This underlines the important role that the assessment of personality and associated positive psychological variables can play in the design of adequate psychological counseling in breast cancer units, optimizing the provision of healthcare resources during the cancer experience [54].

### 4.1. Study Limitations and Future Research

This study has three main limitations. First, the participants were recruited by convenience sampling through a single cancer association, thus limiting the generalizability of the findings. Second, the cross-sectional design and correlational nature of the data means that no conclusions can be drawn regarding causal relationships between personality traits and psychological adjustment. Thirdly, information about the treatments that participants were exposed to has not been considered. Future studies should include this information in order to understand its impact on psychological adjustment. It would also be interesting to study how personality traits are related to other psychological variables (e.g., stress, resilience, coping strategies, etc.) and how these traits can mediate or moderate the relation between stressors and health outcomes in breast cancer patients.

### 4.2. Clinical Implications

The MCMI assesses a continuum from normal personality traits to severe personality disorders, in addition to assessing relationships with clinical syndromes. Its use here has enabled us to identify two groups of breast cancer patients with a distinct personality profile: one group characterized by more psychological vulnerability and another by greater psychological adjustment. The vulnerable group corresponds to patients at risk of psychological maladjustment and who are therefore a target for psychological and social support. By contrast, the psychologically adjusted group corresponds to patients with a more positive outlook who are less likely to experience anxiety or intense emotional distress during the disease process and hence have different psychological needs. 

These results suggest that, within any given group of cancer patients, different kinds of psychological support could be offered based on an assessment of specific needs. There would also be a role here for peer support, which has been shown to be associated with well-being and psychological adjustment in the experience of breast cancer [55]. In this context, Legg et al. [56] described two peer support prototypes among women with breast cancer: needy and resilient. In conjunction with our findings, this suggests that a psychologically adjusted or resilient group of patients may be able to offer support to their more vulnerable peers who are having a similar cancer experience. 

## 5. Conclusions

Our study shows that an assessment of personality traits among women with breast cancer may help to identify patients at risk of psychological distress and, therefore, provide a platform on which to design appropriate interventions to prevent the development of clinical syndromes. Positive psychological factors appear to protect against psychological maladjustment, helping patients to deal more effectively with the experience of cancer.

## Figures and Tables

**Table 1:** Sample characteristics (*n* = 109).

Variables	Percentage
Marital status	
Married	62.4
Single	22.0
Divorced	7.3
Widowed	8.3
Educational level	
Primary	44.0
Secondary	27.5
University	28.5
Age at diagnosis	
<50	57.8
50–69	39.4
≥70	2.8
Time since diagnosis	
<2 years	49.9
2–5 years	27.2
>5 years	22.9
Breast cancer stage	
0	4.6
I	8.3
II	55.0
IIIA	23.9
IIIB	4.6
IIIC	2.8
IV	0.8

**Table 2:** Mean base rate (BR) scores on the personality scales (standard deviation in brackets) for the two groups (clusters) and value of *t*-test (or Welch’s *t*-test) and Cohen’s *d*.

Personality Scales	Group 1 (*n* = 39)		Group 2 (*n* = 63)		*T*	*d*
Schizoid ^a^	53.21	(18.18)	34.86	(21.61)	4.60 ***	0.92
Avoidant	56.59	(20.20)	23.08	(19.04)	8.44 ***	1.71
Depressive (Melancholic)	57.23	(17.51)	17.95	(14.97)	12.05 ***	2.41
Dependent	61.08	(19.87)	32.11	(18.42)	7.48 ***	1.51
Histrionic ^a^	53.05	(19.29)	68.44	(13.14)	−4.40 ***	−0.93
Narcissistic ^a^	50.38	(19.92)	63.00	(9.82)	−3.69 ***	−0.75
Antisocial ^a^	54.38	(10.51)	27.49	(21.37)	8.47 ***	1.60
Sadistic ^a^	57.38	(11.83)	25.70	(21.09)	9.71 ***	1.85
Compulsive	59.92	(10.86)	73.16	(14.38)	−4.94 ***	−1.04
Negativistic ^a^	61.46	(11.58)	28.02	(17.91)	11.45 ***	2.21
Masochistic	48.33	(12.78)	15.49	(14.65)	11.54 ***	2.39
Schizotypal	51.90	(15.64)	21.41	(20.42)	7.98 ***	1.68
Borderline	53.44	(15.26)	15.11	(13.34)	13.34 ***	2.67
Paranoid	63.90	(9.54)	24.79	(22.40)	12.18 ***	2.27

^a^ Welch’s *t*-test. *** *p* < 0.001.

**Table 3:** Adjusted means (standard error in brackets) on the clinical syndrome scales, *F*-statistics and partial eta squared (time since diagnosis as covariates).

Clinical Syndrome Scales	Vulnerable Group (*n* = 39)	Psychologically Adjusted Group (*n* = 63)	*F*	η^2^_p_
Anxiety	78.39 (4.12)	34.58 (3.21)	68.07 ***	0.41
Somatoform	63.90 (3.99)	29.52 (3.10)	44.85 ***	0.31
Bipolar (Manic)	61.92 (3.47)	35.65 (2.71)	34.25 ***	0.26
Dysthymia	65.66 (3.59)	20.43 (2.80)	95.41 ***	0.49
Alcohol Dependence	57.06 (3.21)	38.23 (2.50)	20.72 ***	0.17
Drug Dependence	55.44 (3.38)	26.25 (2.64)	44.83 ***	0.31
Post-Traumatic Stress Disorder	57.55 (3.32)	24.39 (2.58)	60.12 ***	0.38
Thought Disorder	52.57 (2.70)	22.39 (2.10)	74.84 ***	0.43
Major Depression	57.91 (3.24)	21.07 (2.53)	77.62 ***	0.44
Delusional Disorder	51.38 (4.87)	24.31 (3.80)	18.56 ***	0.16

*** *p* < 0.001.

**Table 4:** Adjusted means (standard error in brackets) on the measures of optimism and well-being, *F*-statistics, and partial eta squared (time since diagnosis as covariates).

Optimism and Well-Being	Vulnerable Group	Psychologically Adjusted Group	*F*	η^2^_p_
Optimism ^a^	4.48 (0.45)	6.76 (0.35)	15.50 ***	0.15
Life satisfaction ^b^	19.84 (0.82)	23.81 (0.65)	13.79 ***	0.13
Positive affect ^c^	16.81 (0.71)	19.81 (0.56)	10.31 ***	0.10
Negative affect ^c^	16.08 (0.68)	12.69 (0.54)	14.54 ***	0.13

^a^*n* groups: 36 vs. 56; ^b^ 37 vs. 59; ^c^ 39 vs. 61, respectively. *** *p* < 0.001.
